# Prospective Study of Stereotactic Body Radiation Therapy for Thymoma and Thymic Carcinoma: Therapeutic Effect and Toxicity Assessment

**DOI:** 10.1038/s41598-017-12909-z

**Published:** 2017-10-19

**Authors:** Xue-jun Hao, Bo Peng, Zejun Zhou, Xue-qin Yang

**Affiliations:** 1 Cancer Center, Daping Hospital, Third Military Medical University, Chongqing, 400042 China; 2 Medical center, Medical team, The 68261 corp, Yinchuan, 750024 China

## Abstract

Stereotactic body radiation therapy (SBRT) is an important modality in treatment of tumors. We hypothesized that SBRT can achieve excellent local control with limited toxicity in patients with thymic tumors. A single-institution prospective study was performed with 32 patients who underwent SBRT of thymoma and thymic carcinoma between 2005 and 2014. Thirty-two patients including 39 target lesions were analyses in this study. Almost half of the patients (46.9%) were type C by histopathology and more than half (56.3%) were classified into stage IVA or IVB. The median dose of SBRT for gross tumor volume (GTV) was 56 Gy (range 49–70 Gy). Results showed that the response rate was 96.9% after SBRT and the median tumor shrinkage rate was 62.2% (range 3.8–100%). For the patients with both stage II–III and type A-B (n = 6), the median PFS was 28 months. In-field failure was only observed in 4 patients, and outside-field failure was seen in 2 patients. The local control rate was 81.25%. Patients treated with SBRT had an excellent local control with mild toxicities, which suggests that SBRT is feasible for the patients with thymic tumors who are unable to undergo either surgery or conventionally fractionated radiation therapy.

## Introduction

Thymoma is mainly divided into two types, invasive and noninvasive type, and more than 60% of patients belong to noninvasive thymoma^1^. Thymic carcinoma is a rare cancer that is more aggressive and shows a poorer prognosis compared with thymoma. When feasible, complete surgical resection is the primary treatment.

There are quite a number of thymoma patients that has lost its surgical indications when diagnosed. Even after complete resection, the recurrence rate can be about 20%^2^. For patients with unresectable or recurrent disease, radiation is routinely administered, often in combination with systemic chemotherapy^3,4^. However, because of wide range of radiation, more complications of conventional radiotherapy limit its treatment dose^5,6^. The local recurrence rates of conventional radiotherapy are up 16% to 45%^7,8^. Moreover, it’s not suitable for patients with severe heart and lung disease. Stereotactic body radiation therapy (SBRT) has been extensively applied in a variety of solid tumors such as lung cancer and liver cancer, predominantly in the medically inoperable, where it has been shown to have high local control rates and has low toxicities^9,10^. On the one hand, by improving the single dose, SBRT not only shortens the total radiation treatment, but also increases the total dose of equivalent biological effects^11^; On the other hand, SBRT, with more precise conformal radiation therapy, significantly reduces the damage to the surrounding normal tissues^12^.

There are five studies with 19 cases of SBRT for thymoma reported at present and the results showed that SBRT for thymoma was effective as well with low toxicities^13–17^. However, the number of the cases is too small and, no prospective studies reported until now. Here we performed a prospective study on the treatment of thymoma by SBRT. The purpose of this prospective study was a preliminary evaluation of feasibility, efficacy and toxicity of γ-SBRT in the treatment of thymic tumors.

## Results

### Clinicopathological characteristics of patients

Thirty-two consecutive patients including 39 target lesions were analyzed in this study. All the patients have completed the treatment. Patient characteristics are described in Table 1. The median age of the patients was 51 years (range 23–76 years) and 18 of these patients were male. The median tumor diameter was 5.0 cm (range 1.5–12.3 cm). Almost half of the patients (46.9%) were type C by histopathology and more than half (56.3%) were classified into IVA or IVB. There are 7 cases of Masaoka stage IV in the 15 patients with type C (46.7%). Thirteen patients have myasthenia gravis symptoms. The median dose of SBRT for GTV was 56 Gy (range 49–70 Gy). Nineteen patients chose to perform chemotherapy after SBRT. In this study, there were eighteen patients that didn’t accept any kind of surgery, especially three patients with stage II. However, among them, the patient with type C developed metastasis quickly after SBRT without chemotherapy as the patient refused, and the other two patients with type B acquired completed response after single SBRT therapy, thus they didn’t need any surgery (Table 1 and supplemental material).

**Table 1:** Demographic and baseline characteristics of the study participants (N = 32).

**Variable**	**N(%)**
**Gender**	
Male	18 (56.3)
Age (years)	
Median	51
Range	23–76
**Symptomsat presentation**	
Myasthenia gravis	13 (40.6)
**WHO performance status**	
0	3 (9.4)
1	18 (56.2)
2	11 (34.4)
**Tumor size (cm) (n** = **39)**	
Median (range)	5 (1.5–12.3)
<5 cm	19 (48.7)
<8 cm, ≥5 cm	10 (25.6)
≥8 cm	10 (25.6)
**Histopathology**	
A	0 (0)
AB	1 (3.1)
B1	1 (3.1)
B2	11 (34.4)
B3	4 (12.0.5)
C	15 (46.9)
**Modified Masaoka stage**	
I	0 (0)
II	4 (12.5)
III	10 (31.3)
IVA	8 (25)
IVB	10 (31.3)
**Resection status**	
None	19 (59.4)
Biopsy by surgery	1 (3.1)
Incomplete resection	1 (3.1)
Recurrence after resection	10 (31.3)
Preoperative	1 (3.1)
Combination chemotherapy	19 (59.4)
**Gross tumor volume (Gy)**	
Median (range)	56 (49–70)

### Univariate analyses in the modeling group

**Figure 1:**
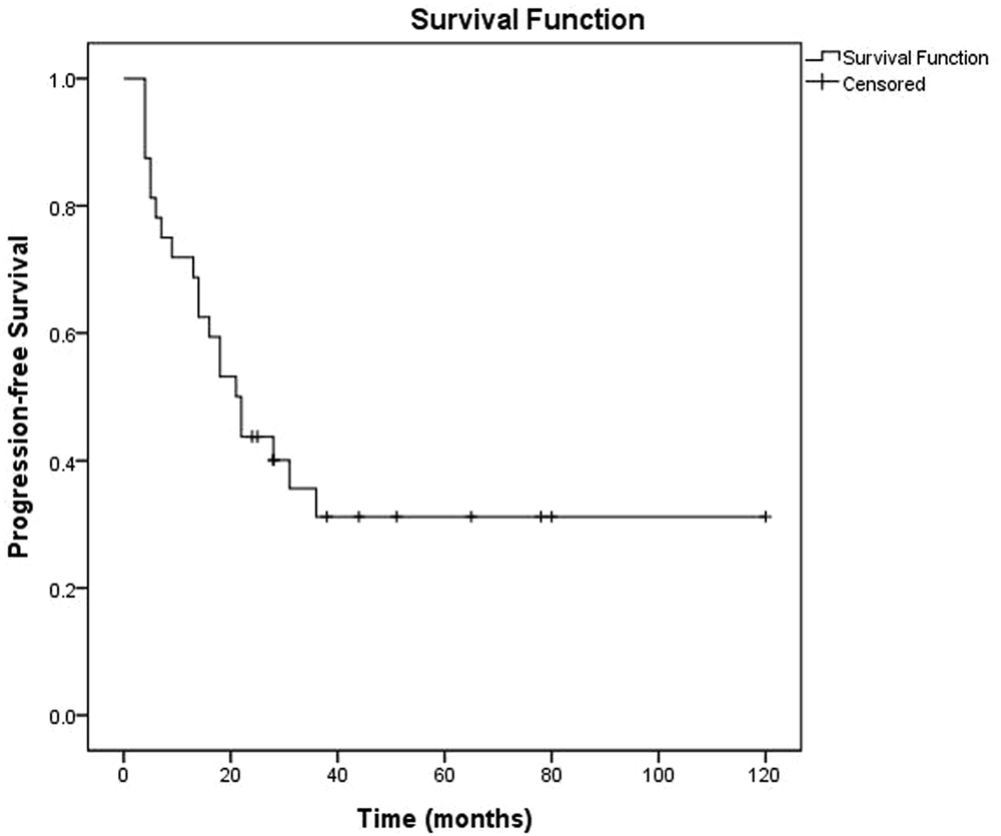
Kaplan–Meier curve for progression-free survival (PFS) of patients with thymoma (n = 32).

**Figure 2:**
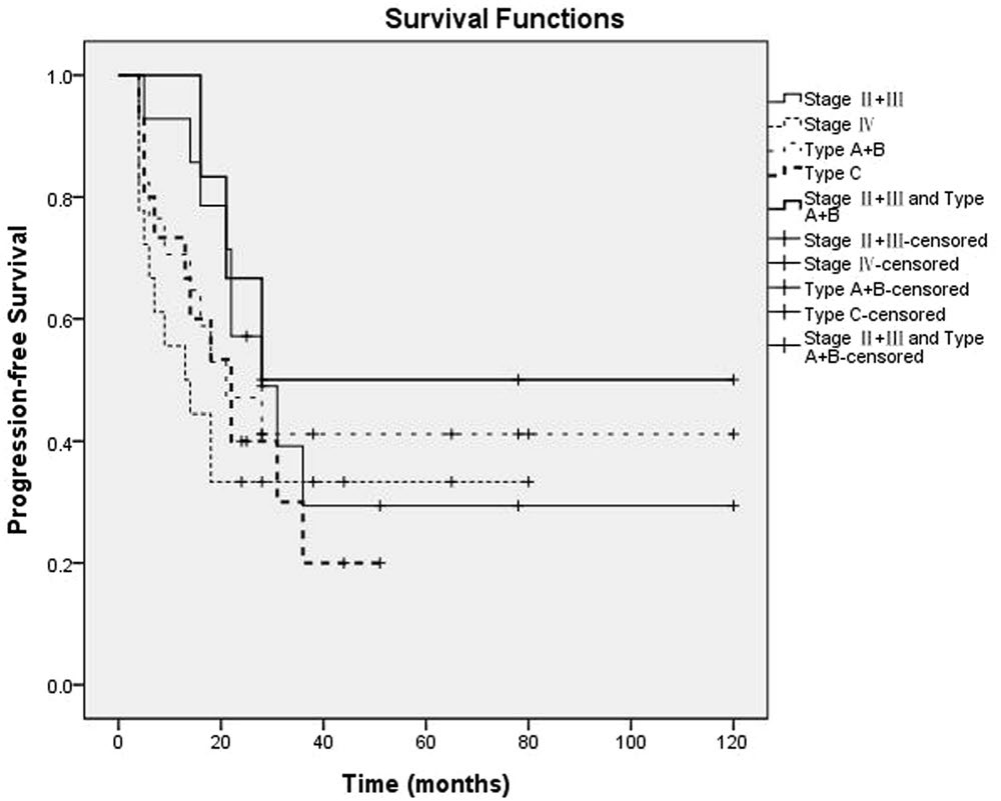
Kaplan–Meier curve for progression-free survival (PFS) of patients with different stage and type.

All patients were followed up until death or this article published before. The median follow-up time is 54 months (range 26–128 months). Until the last follow up, all but six patients (two with type B and four with type C) were still survival until the last follow up. The median PFS was 21 months (95% CI 14.3–27.7 months) (Fig. 1). Median PFS of II- III (n = 14) was 28 months (95% CI 14.6–41.4 months) and median PFS of IV (n = 18) was 13 months (95%CI 2.6–23.4 months). Based on different histopathology type the median PFS of type A-B (n = 17) was 21 months, and median PFS of type C (n = 15) was 22 months. For the patients with both stage II- III and type A-B (n = 6), the median PFS was 28 months (Fig. 2).

**Figure 3:**
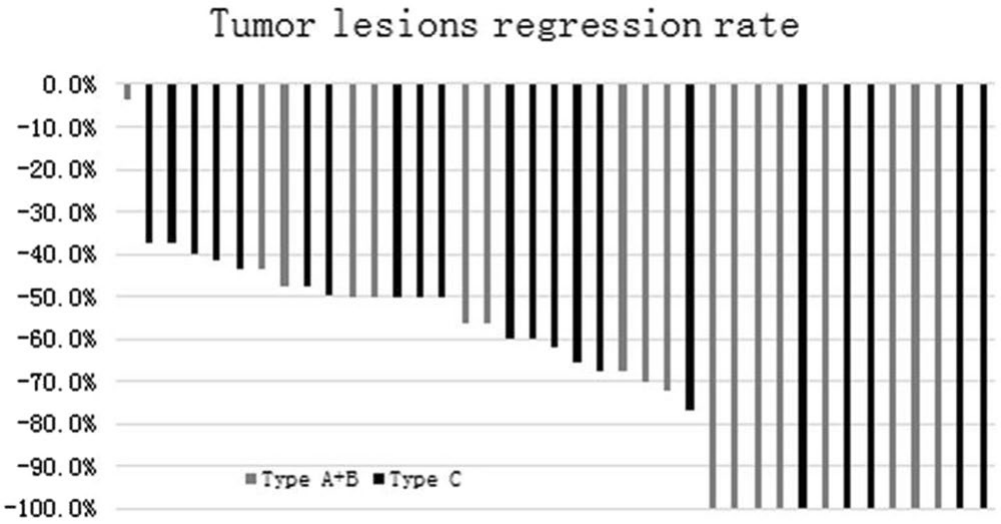
Tumor lesions regression rate.

Due to tumor shrinkage usually occurred half a year after SBRT treatment of thymoma, the evaluation time of tumor response was designed at the time of 3 months after SBRT. Results showed that 11 (34.4%, 11/32) patients achieved CR, 20 (62.5%, 20/32) patients achieved PR, and 1 (3.1%, 1/32) patients with type B had SD. Among the patients with CR, Four belonged to type C (see supplemental material). The estimated objective response rate was 96.9% after SBRT. The average tumor size was 1.9 cm (range 0–6.2 cm) after SBRT, which was markedly smaller than the baseline tumor size (average 5.3 cm; range 1.5–12.3 cm; t = 10.576, P = 0.000). The median tumor shrinkage rate was 62.2% (range 3.8–100%) (Fig. 3). Median tumor shrinkage rate of type A-B (n = 18) was 71% (3.8–100%) and median tumor shrinkage rate of type C (n = 21) was 60% (37.1–100%), there was no statistically significant difference on tumor shrinkage rate (p = 0.262; t = 1.14). Myasthenia gravis remission rate was 100% (13/13).

### Relapse status and toxicity assessment

**Figure 4:**
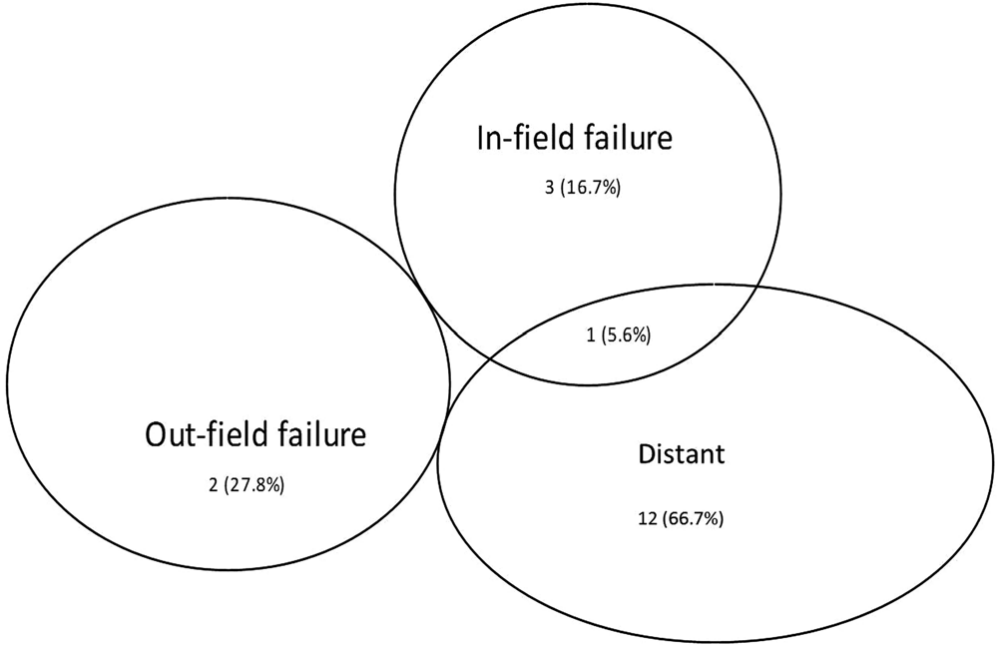
Patterns of tumor recurrence in the study participants (n = 18).

Tumor recurred in the majority (18/32, 56.3%) of the patients. However, tumor recurrence is associated with Masaoka staging and histopathology type. There were 11 cases of recurrence in the 18 patients with stage IV (61.1%) and 10 patients in the 15 patients with type C (66.7%). Among the recurrence patients, most (66.7%, 12/18) were simple distant recurrence patients. Only in-field recurrence was observed in 3 (16.7%, 3/18) patients. One patient had both in-field and distant metastasis. Simple out-of-field recurrence was seen 2 (27.8%, 2/18) of patients (Fig. 4). Thus the local control rate was 81.25%.

The radiation-induced side effects were mild, with most grades 1–2 and no grade 3–4. None of the patients discontinued treatment due to radiation toxicities. Main adverse reaction was radioactive esophagitis with 8 (8/32, 25%) cases of grade 1 and 3 (3/32, 9.4%) cases of grade 2. Others included neutropenia, fatigue, anorexia, dermatitis and nausea/vomiting, which were mild and tolerable. Grade 1 and grade 2 pneumonitis was seen in 15.6% (5/32) of the patients occurred within 90 days of the last radiation. No severe late toxicity to centrally located structure was observed. Toxicity was detailed in Table 2.

**Table 2:** Adverse event within the first 4 weeks.

Toxicity	Grade 1 (%)	Grade 2 (%)	Grade 3 (%)	All grades (%)
**Hematologic**				
Anemia	0 (0)	0 (0)	0 (0)	0 (0)
Neutropenia	4 (12.5)	1 (3.1)	0 (0)	6 (18.8)
Thrombocytopenia	1 (3.1)	0 (0)	0 (0)	0 (0)
**Non-hematologic**				
Fatigue	4 (12.5)	0 (0)	0 (0)	4 (12.5)
Dermatitis	4 (12.5)	0 (0)	0 (0)	4 (12.5)
Anorexia	7 (21.9)	1 (3.1)	0 (0)	8 (25)
Nausea/vomiting	5 (15.6)	2 (6.2)	0 (0)	7 (21.9)
Esophagitis	8 (25)	3 (9.4)	0 (0)	11 (34.4)
Pneumonitis	^*^4 (12.5)	^*^1 (3.1)	0 (0)	5 (15.6)

^*^Evaluate within 3 months.

## Discussion

At present, only five literatures have reported 19 cases of SBRT for thymoma, and the most of these reports are case reports^13,15,16^. This is the first cohort study investigating the use of SBRT for treatment of patients with thymic tumors. We demonstrate excellent target coverage with low doses of radiation delivered to ORAs. This early, exploratory analysis demonstrates that patients treated with SBRT had low rates of acute toxicities and excellent local control, which suggests that SBRT is feasible for patients who are unable to undergo either surgery or conventionally fractionated radiation therapy, or as the palliative therapy for the metastases of thymic tumor.

Radiation therapy (RT) plays an important adjuvant role in the treatment of invasive thymoma (ie, stage II and higher disease). Mayer and colleagues^18^ presented small series data evincing a potential relationship between dose delivered and local control probability. Most of studies proposed the prescription dose of 40–60 Gy/20–30 fraction^19–21^, administered in 4–6 weeks, by using 1.8–2 Gy daily, 5 days per week. For patients with incomplete resection of thymoma, the radiation dose currently used for thymoma is 45–50 Gy for clear or close resection margins, 54 Gy for microscopically positive resection margins, and 60 Gy for grossly positive margins. In the case of inoperable disease or bulky gross residual tumor, a dose of 40–45 Gy was given followed by boost of 20 Gy. A phase II intergroup study was performed using four cycles of cisplatin-doxorubicin-cyclophosphamide chemotherapy and 54 Gy RT to the primary and nodal regions^22^. The results in 23 patients demonstrated a high response rate of 69.6%. Zhu G and colleagues^23^, in a series of adjuvant and definitive radiotherapy treated thymoma patients, noted 51–77% local control rates with resection.

The prominent characteristics of SBRT can be summarised as higher precision, higher target dose, higher target conformity and delivery in a lower number of fractions. In the present study, a total dose of 35–50 Gy was delivered to the 50% isodose line covering 100% of the PTV, with 49–70 Gy to the 70% isodoseline covering 95% of the GTV. The biological equivalent dose (BED, α/β = 10) regarding the GTV was 73–119 Gy, which represents an obvious increase dose as compared with conventional radiotherapy. Treatment time is generally completed within 2 weeks (no more than 17 days), compared with the conventional radiotherapy 4–6 weeks of treatment, which shorten the total treatment time, and further improve the equivalent biological dose. Theoretically, the local control rate should be much higher than the conventional radiotherapy. Actually, in the study reported by Fan *et al*.^11^, they found that, compared with 40 patients who underwent conventionally radiation therapy (45–70 Gy in 25–40 fractions), 5 patients who underwent SBRT (65–70 Gy in 8–10 fractions) experienced a longer median survival duration (54 months vs. 44 months). In this study, the tumor response rate was 96.9% and tumor regression was obvious. Average tumor size was 1.9 cm after SBRT and it was markedly smaller than the baseline tumor size (average 5.3 cm; t = 10.576, P = 0.000). These data suggested that SBRT had a preferably local control rate.

As we know, this study only enlarged the radiation field 0.5–1 cm outside the GTV. Also the PTV didn’t include the nodal regions. Although tumor recurred in the majority (56.3%, 18/32) of the patients, tumor recurrence was associated with Masaoka staging and histopathology type. Moreover, most (12 (66.7%)) were distant recurrence. Outside-field failure was only observed in two patients. Zhu and colleagues^23^ reported that no local control benefit with regard to extended field techniques and increased incidence of pneumonic injury, attributable to increased volumes of lung and heart tissue in the extended field volume.

The early studies reported that grade 3–4 toxicity of radiation ranged from 11% to 13% in conventional radiotherapy^24,25^. With the improvement of radiotherapy technology, a decrease in such toxicities (5% to 10%) had been reported in recent years^26^. Vogel reported much lower toxicities treated by double-scattering proton beam therapy (DS-PT) with acute grade 2 toxicities included dermatitis (37%), fatigue (11%), esophagitis (7%), pneumonitis (4%) and no patient experienced grade ≥3 toxicity^27^. In this study, we didn’t find any grade 3–4 toxicities of SBRT and grade 2 toxicities were also few. Main adverse reaction regarded to thymus lesions was radioactive esophagitis (11/32, 34.4%). The mild toxicities of SBRT may be due to the unique dose superiority of SBRT which is characterized by a very sharp dose fall off that offers greater sparing of critical normal tissue, relative to conventional radiotherapy, which suggested that SBRT for thymic tumor may be more suitable for the patients with chronic obstructive pulmonary emphysema and other severe heart and lung diseases.

Compared to our γ-SBRT system, CyberKnife with 6-MVX radiation should be the most widely used SBRT system in the world. SBRT with CyberKnife requires a highly conformal technique to deliver high-dose radiation in 5 or fewer treatment fractions, it is characteristic of less fraction and lower radiation effect to the surrounding organs. Harada Y. *et al* ^17^. reported ten patients of thymoma treated by CyberKnife. The dose ranged from 31 to 50 Gy, and the fraction ranged from 7 to 12 times. Two patients achieved CR, and the ORR was 90%. The side effects were minimal. Only four patients had mild radiation pneumonia that did not require treatment. This study suggested that SBRT with CyberKnife was successful in patients with primary tumor, relapse, and metastatic lesions of thymic carcinoma. However the prescription dose ranged too large in different patients. Thus more CyberKnife prospective studies are needed to guide clinical applications. The prospect of CyberKnife treatment would have immense potential clinical impact.

Our study is limited by the patient numbers and short follow-up. The population of patients treated was heterogeneous, including patients receiving definitive radiation therapy, salvage radiation therapy, and others with a variety of systemic chemotherapy regimens and radiation doses. Given the rarity of thymic tumor, homogeneous patient populations with long term follow-up are limited in single institutions. Further study from multi-national databases such as the International Thymoma Interest Group International Registry may contribute to a better understanding of both the role of radiation therapy and the differences in long-term toxicities between radiation modalities^28^.

## Methods

### Study design and patients population

The clinical data of the patients with thymic tumors who had received SBRT between January 2005 and January 2014 were prospective evaluated. Histopathology was defined by the 2004 WHO classificatiion system^1^. Staging was assigned based on review of operative and pathology reports based on the Masaoka system^29^.

Patient selection criteria: we recruited patients with thymic tumors starting from the study. Criteria for enrollment in the study were as follows: (1) the age of patients was ≥18 years; (2) their performance status according to WHO was 0–2; (3) histology and pathology diagnosis of thymoma; (4) patients with assessment measurable lesion; (5) informed patient consent obtained for SBRT; The exclusion criteria were as follows: (1) have a mental illness; (2) vital organs have a serious illness or serious infection; (3) patients are other new drug tests or enter the study within 1 month before the other new drug clinical trials; (4) pregnant or lactating women or in the growth period of patients who were not using contraception.

### Therapeutic method

**Figure 5:**
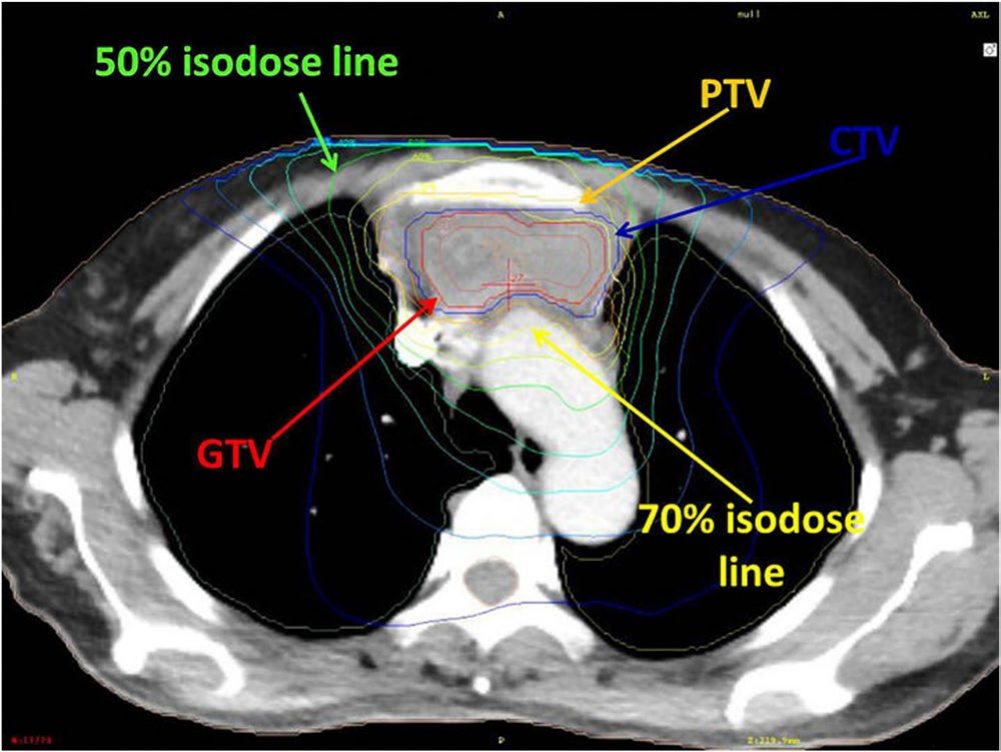
Treatment plan for thymoma (n = 32).

All patients were placed in the supine position or prone position with a thermoplasticbody cast combined with a vacuum pillow during treatment planning and SBRT. CT images for treatment planning were obtained under normal breathing and with breath holding during the expiratory and inspiratory phases. The planning CT was scanned with 3 mm slice throughout the tumor, and 5 mm slice in other areas of the thorax and upper abdomen. After the scan was finished, the positional parameters were recorded in order to repeat the position when the patient was irradiated. Then CT images were transferred to the treatment planning system (OUR WB-GR TPS99) via the internet. Treatment planning was then performed in the same position, using slow-rotation serial CT scanning. The gross target volume (GTV) on CT during the three phases was superimposed on the 3D radiation treatmentplanning system and was delineated in the lung window on CT image. An additional margin of 0.5 cm in the axial plane and 1.0 cm in the longitudinal from GTV was used to delineate the planning target volume (PTV). The clinical target volume (CTV) was defined as the surrounding area of the GTV plus at most 5 mm in all directions considering the OAR. If an ORA was very close to the GTV, the CTV was usually decreased manually, or the GTV was treated as CTV. Rotation focus beams were generated by the gamma-ray treatment system, and the target fusion technique was used to fit the target shape. A total radiation dose of 35–50 Gy was delivered to the 50% isodose line covering at least 95% of the PTV (3.5–5 Gy/fraction), and a total radiation dose of 49–70 Gy was delivered to the 70% isodose line covering at least 95% of GTV (4.9–7 Gy/fraction) (Fig. 5). Treatment doses and fractions in SBRT were dependent on tumor size and proximity to critical structures. SBRT was delivered in ten fractions over 2 weeks (Table 2). At the time of treatment planning, the dose delivered to critical structures and adjacent of organs, including the main bronchi, esophagus, trachea, heart, and major blood vessels, was confirmed to be <50 Gy (5 Gy/fraction), and the dose delivered to the spinal cord was confirmed to be <30 Gy (3 Gy/fraction)^30–32^.

Whether chemotherapy or not was determined by patients themselves. Chemotherapy was performed after 1 month.

### Follow-up and effect assessment

After completion of γ-SBRT, all of the patients were regularly examined until the last clinical follow-up. Follow-up included medical history, physical examination, blood work and a CT scan. Chest CT scan was performed one and three months after SBRT, and then every 3 months for 2 years, and every 6 months thereafter. CT examination of the chest was performed to identify local recurrence or distant metastasis. A bone scan or PET was performed if necessary. The information regarding recurrence, toxicity and survival were recorded via telephone, mail and local hospital imaging reports.

Clinical effects were classified on the basis of single diameter measurement and solid tumor response evaluation criteria as complete remission (CR), partial remission (PR), stable disease (SD) and progressive disease (PD) according to the CT images. CR+ PR were considered as an effective evaluation. Vital signs and adverse events were monitored throughout the study. Safety evaluation was based mainly on the occurrence, frequency, and severity of adverse events, which were graded weekly according to the common terminology criteria for adverse events version of the radiation therapy oncology group (RTOG). Patients were assessed on a daily basis during SBRT and a weekly basis until acute grade 2 toxicity had resolved and subsequently every 3 months. Death was recorded when experienced.

### Statistical analysis

All analyses were done using the SPSS statistical package (ver.18.0) (SPSS Inc. Chicago, IL, USA). All reported P values were two-sided, and a significance level = 0.05 was used. A p-value < 0.05 was considered statistically significant. Comparison of the thymic tumor size of 39 target lesions before and after γ-SBRT was analyzed using the paired t test. The Kaplan–Meier curve and lifetables were used to describe survival data. Nonparametric confidence interval (CI) was calculated for the medians, and 95% CIs were constructed. All survival results were based on full data analysis; for patients who withdrew or were lost to follow-up, the last observation carried forward was used. Safety analysis was based on the safety sets defined as all patients who received at least one fraction of the radiation and had at least one follow-up safety assessment. Adverse events were analyzed mainly using descriptive statistics. Shrinkage rate was defined as the ratio of the decrease in the longest diameter of target lesions to the baseline tumor size. PFS measured from the start of SBRT until any area in recurrence or distant metastasis. Local recurrences were then subclassified as occurring in-field (encompassed entirely within the 70% isodose line), out-of-field or marginal (relapse occurring partially or fully in the penumbra, commonly between the 20% and 70% prescription isodose lines) according to the relationship of the tumor recurrence to the prescription isodose line.

### Ethics approval

All patients with thymoma or thymic carcinoma requiring radiation therapy treated with SBRT. This study was approved by the Ethics Committee of the Daping Hospital, Third Military Medical University, and was performed in accordance with the ethical standards laid down in the 1964 declaration of Helsinki and all subsequent revisions. All persons mentioned in the paper gave their informed consent prior to inclusion in the study.

ClinicalTrials.gov ID (Identifiers): NCT03078699, Date of registration: 03/07/2017.

## Electronic supplementary material

supplemental information
