# Supplementary material for: Prospective Study of Stereotactic Body Radiation Therapy for Thymoma and Thymic Carcinoma: Therapeutic Effect and Toxicity Assessment
Source: Sci Rep. 2017 Oct 19;7:13549. doi: 10.1038/s41598-017-12909-z (PMC5648828; doi:10.1038/s41598-017-12909-z)
Supplement: Supplementary file 1 — supplemental information [file 41598_2017_12909_MOESM1_ESM.docx]

Prospective Study of Stereotactic Body Radiation Therapy for Thymoma and Thymic Carcinoma: Therapeutic Effect and Toxicity Assessment (supplementary information)

Xue-jun Hao ^1,2,#^, Bo Peng^1,#^, Zejun Zhou^1^, Xue-qin Yang ^1*^

***Corresponding Author:** Xueqin Yang, Cancer Center, Daping Hospital, Third Military Medical University, No.10 Changjiang Zhi lu, Daping Yuzhong District, Chongqing 400042, China. Tel: +86-23-68757151, Fax: +86-23-68611206, E-mail: [yangxueqin@hotmail.com](mailto:yangxueqin@hotmail.com).

# These two authors contributed equally to this work.

|  | Stage | WHO  histology | Resection  status | Tumor  Size  (cm) | Treatment  sites | Chemotherapy | Dose for GTV  (Gy) | Effect evaluation |
| --- | --- | --- | --- | --- | --- | --- | --- | --- |
| 1 | Ⅲ | C | Recurrence after resection | 5 | Thymus | No | 63 | PR |
| 2 | Ⅲ | C | No | 8 | Thymus | No | 56 | PR |
| 3 | Ⅱ | B2 | No | 2.1 | Thymus | No | 70 | CR |
| 4 | Ⅲ | B3 | No | 7.5 | Thymus | No | 56 | PR |
| 5 | Ⅱ | C | No | 4.6 | Thymus | No | 56 | PR |
| 6 | ⅣB | B1 | Recurrence after resection | 5.2 | Mediastinum | No | 56 | CR |
| 7 | Ⅲ | B2 | No | 8 | Thymus | Yes | 56 | CR |
| 8 | Ⅱ | B3 | No | 1.5 | Thymus | No | 56 | CR |
| 9 | ⅣB | C | No | 2.5 | Liver | No | 56 | CR |
| 10 | ⅣB | C | No | 5.1 | Thymus | No | 49 | PR |
| 11 | ⅣB | B3 | Postoperative residual | 2.2 | Mediastinum | Yes | 56 | CR |
| 12 | ⅣB | B2 | Recurrence after resection | 4 | Mediastinum | Yes | 56 | PR |
| 13 | ⅣB | B2 | No | 9 | Mediastinum | Yes | 56 | PR |
| 14 | ⅣB | C | No | 3 | Right lung | Yes | 63 | PR |
|  |  |  |  | 2 | Liver |  | 63 |  |
|  |  |  |  | 7.4 | Thymus |  | 56 |  |
| 15 | ⅣA | C | No | 3 | Pleura | Yes | 56 | PR |
|  |  |  |  | 12.3 | Thymus |  | 49 |  |
| 16 | Ⅲ | C | Recurrence after resection | 3.2 | Thymus | Yes | 70 | CR |
| 17 | ⅣB | C | Recurrence after resection | 6 | Mediastinum | Yes | 56 | PR |
|  |  |  |  | 3 | Pleura |  | 56 |  |
|  |  |  |  | 3 | Liver |  | 56 |  |
| 18 | Ⅲ | C | No | 7.5 | Thymus | Yes | 49 | PR |
| 19 | ⅣA | B2 | No | 9.1 | Thymus | Yes | 49 | PR |
| 20 | ⅣA | B2 | Recurrence after resection | 3.2 | Pleura | Yes | 56 | PR |
| 21 | Ⅱ | B2 | Preoperative | 6.5 | Thymus | Yes | 53.2 | PR |
| 22 | ⅣA | A or AB | No | 8 | Thymus | Yes | 53.2 | PR |
| 23 | Ⅲ | C | No | 7 | Thymus | No | 63 | PR |
| 24 | ⅣA | B2 | No | 1.9 | Pleura 1 | Yes | 70 | CR |
|  |  |  |  | 2.2 | Pleura 2 |  | 70 |  |
| 25 | ⅣA | B2 | Recurrence after resection | 8.6 | Pleura | Yes | 49 | CR |
| 26 | ⅣB | C | No | 5.8 | Thymus | Yes | 56 | PR |
|  |  |  |  | 3.2 | Mediastinum |  | 56 |  |
| 27 | Ⅲ | C | No | 8.6 | Thymus | Yes | 49 | PR |
| 28 | ⅣA | B2 | Recurrence after resection | 2.6 | Pleura | Yes | 49 | SD |
| 29 | ⅣA | B2 | Biopsy by surgery | 10 | Thymus | Yes | 56 | PR |
| 30 | Ⅲ | B3 | No | 8 | Thymus | No | 49 | PR |
| 31 | Ⅲ | C | Recurrence after resection | 1.8 | Thymus | No | 63 | CR |
| 32 | ⅣB | C | Recurrence after resection | 3.8 | Mediastinum | No | 49 | CR |

**Table 1:** Treatment of patients with related data


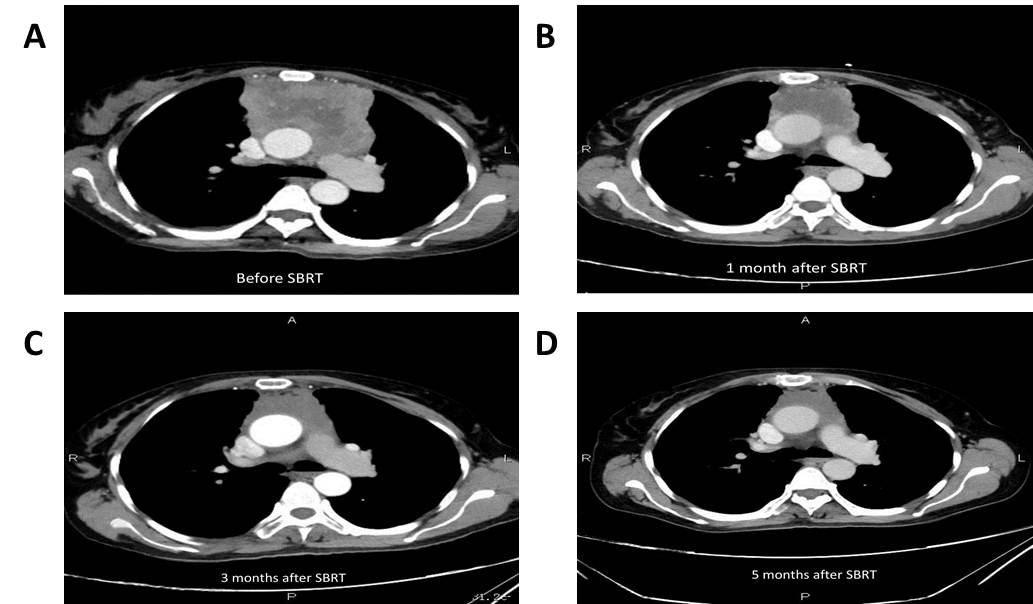


**Figure:** A 65 year-old Masaoka stage Ⅲ thymic carcinoma patient was treated before and after SBRT

1. Thymic carcinoma at presentation prior to SBRT. (B) A month after SBRT of thymic carcinoma. (C) Three months after SBRT of thymic carcinoma. (D) Five months after SBRT of thymic carcinoma.
